# Cytoprotective Mechanisms of DJ-1: Implications in Cardiac Pathophysiology

**DOI:** 10.3390/molecules26133795

**Published:** 2021-06-22

**Authors:** James N. Tsoporis, Ioannis-Alexandros Drosatos, Sahil Gupta, Hajera Amatullah, Shehla Izhar, Claudia C. dos Santos, Vasileos Salpeas, Angelos G. Rigopoulos, Ioannis K. Toumpoulis, Andreas S. Triantafyllis, Eleftharios Sakadakis, Nikolaos Kavantzas, John C. Marshall, Ioannis K. Rizos, Thomas G. Parker

**Affiliations:** 1 Keenan Research Centre for Biomedical Science, Li Ka Shing Knowledge Institute, St. Michael’s Hospital, University of Toronto, Toronto, ON M5B 1W8, Canada; Sahil.Gupta@unityhealth.to (S.G.); shehla.izhar@unityhealth.to (S.I.); claudia.dossantos@unityhealth.to (C.C.d.S.); john.marshall@unityhealth.to (J.C.M.); thomas.parker@unityhealth.to (T.G.P.); 2 2nd Academic Department of Cardiology, Attikon University Hospital, University of Athens Medical School, 164 62 Athens, Greece; alexdrosatos@hotmail.com (I.-A.D.); vsalpeas@med.uoa.gr (V.S.); angelos.rigopoulos@gmail.com (A.G.R.); toumpoul@otenet.gr (I.K.T.); andreas.triantafyllis@gmail.com (A.S.T.); elsakadakis@yahoo.gr (E.S.); ioannis.c.rizos@otenet.gr (I.K.R.); 3 Institute of Medical Science, University of Toronto, Toronto, ON M5G 1V7, Canada; 4 Gastrointestinal Unit and Center for the Study of Inflammatory Bowel Disease, Massachusetts General Hospital and Harvard Medical School, Boston, MA 02210, USA; hajera.amatullah@gmail.com; 5 1st Department of Pathology, School of Medicine, National and Kapodistrian University of Athens, 115 27 Athens, Greece; nkavantz@med.uoa.gr

**Keywords:** DJ-1, antioxidant, heart failure, apoptosis, inflammation

## Abstract

DJ-1 was originally identified as an oncogene product while mutations of the gene encoding DJ-1/PARK7 were later associated with a recessive form of Parkinson’s disease. Its ubiquitous expression and diversity of function suggest that DJ-1 is also involved in mechanisms outside the central nervous system. In the last decade, the contribution of DJ-1 to the protection from ischemia-reperfusion injury has been recognized and its involvement in the pathophysiology of cardiovascular disease is attracting increasing attention. This review describes the current and gaps in our knowledge of DJ-1, focusing on its role in regulating cardiovascular function. In parallel, we present original data showing an association between increased DJ-1 expression and antiapoptotic and anti-inflammatory markers following cardiac and vascular surgical procedures. Future studies should address DJ-1’s role as a plausible novel therapeutic target for cardiovascular disease.

## 1. Introduction

**Figure 1:**
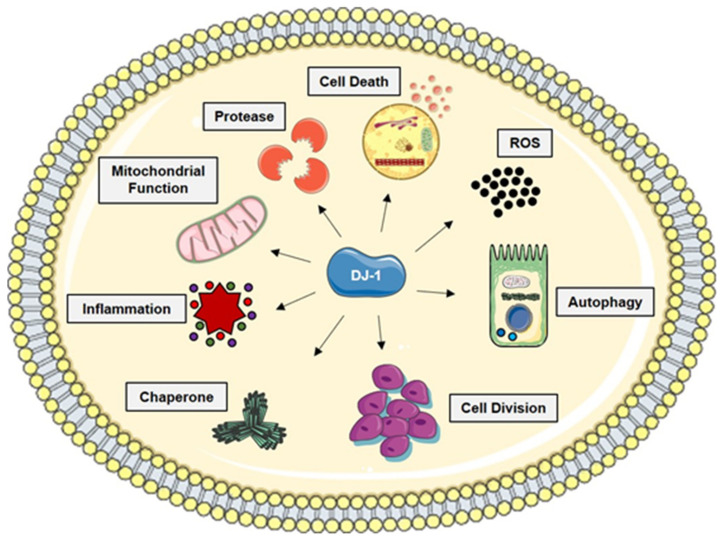
Schematic diagram of the various pathways regulated by DJ-1. Representation of the different DJ-1 activation pathways. DJ-1 possesses chaperone and protease activities and regulates pathways involved in cell death, ROS production, autophagy, cell division, and inflammation.

DJ-1 is a ubiquitously expressed, highly conserved protein that was originally identified as a mitogen-dependent oncogene product involved in a Ras-related signal transduction pathway [1]. In 2003, Bonifati et al. [2] reported that the homozygous deletion or missense mutation of DJ-1 is associated with PARK7, a monogenic form of autosomal recessively inherited early-onset Parkinson’s disease (PD) characterized by neurodegeneration. These findings imply that DJ-1 has a role in cellular homeostasis. Decreased DJ-1 expression can accelerate cellular apoptosis (as in PD) whereas overexpression promotes uncontrolled proliferation (as in neoplastic diseases). To date, DJ-1 is reported to be involved in several biological processes, including transcriptional regulation and control of mitochondrial function, proteolysis, autophagy, and chaperone activity (Figure 1). Regarding its molecular function, DJ-1 enhances Ras-mediated oncogenesis [1], modulates the PTEN/Akt survival pathway [3,4], suppresses Ask1-mediated apoptosis [5], and increases glutathione (GSH) synthesis, Hsp70 [6], and tyrosine hydroxylase [7,8] expression. Recently, DJ-1 has been described as a key histone deglycase that rescues glycation-induced damage [9,10]. When phosphorylated, DJ-1 increases its glyoxalase activity preventing glycation-induced histones mis-regulation and preserving the epigenome landscape and, in the case of cancer cells, sustaining proliferation [11]. More recently, DJ-1 has been shown to mediate the development of multiple drug resistance (i.e., cancer cells) [12] by activating the PTEN/PI3K/Akt/Nrf2 pathway and subsequently upregulating anti-apoptotic genes [13]. However, its role in responding to oxidative stress is most thoroughly described and is hypothesized to be its central function. In this regard, DJ-1 can bind a series of target mRNA molecules, identifying a GG/CC-rich sequence that is sufficient for DJ-1 recruitment. The functional classification of these transcripts suggests how DJ-1 may play roles in coordinating responses to oxidative damage and suppression of cell death [14].

Given its role in PD, most published studies have investigated DJ-1’s functional role in neuron cell lines and brain tissues of animals. As such, the first part of this review will focus on the diverse neuronal cell functions of DJ-1. Due to a scarcity of published data, in the second part of this review, a discussion of the cardiovascular functions of DJ-1 will be supplemented with novel data generated specifically for this review. 

## 2. Structure

DJ-1 is a highly conserved, ubiquitously expressed, 23kDa protein consisting of 189 amino acids. DJ-1 contains 11 β-strands (b1–11) and 8 α-helices (a1–8) with the α/β flavodoxin-like fold found throughout the superfamily [15]. Structurally, DJ-1 features a central-parallel β-sheet that is flanked by α-helices and a β3–4 hairpin on one end and an additional antiparallel three-stranded β-sheet on the other end of the molecule [15]. The C-terminal helix (α8) distinguishes DJ-1 clade proteins from other members of the superfamily that share its core fold and is important for dimerization [15].

Several missense mutations have been identified in DJ-1, including M26I, A104T, D149A, E163K, and L166P [16,17,18,19,20,21,22,23,24]. The L166P mutation leads to severe destabilization and unfolding of DJ-1, resulting in a loss of DJ-1 dimerization. DJ-1^L166P^ is unable to function as a chaperone [23] or a protease [22] and is unable to protect cells from H_2_O_2_-induced cell death [18,24,25]. Other DJ-1 mutations (e.g., A104T, E163K, and D149A) appear to reduce the stability of DJ-1 without causing substantial folding defects or loss of dimerization [21]. The M26I mutation has been reported to affect steady-state levels of DJ-1 [17], probably by increasing turnover rates and/or decreasing dimerization [17,19], thus abrogating the anti-oxidative stress function of DJ-1 [26]. 

## 3. Role in Mitochondrial Function

DJ-1 localizes to the mitochondria following oxidation; mutations of the cysteine residues to alanine block oxidation, and therefore prevent mitochondrial translocation [27]. Translocation is seen early after oxidative insults such as H_2_O_2_ and paraquat, with levels of mitochondrial DJ-1 increasing dose-dependently [28,29]. Interestingly, one study showed DJ-1 mutants (cysteine to serine residues) can also undergo mitochondrial translocation [28]. Furthermore, overexpressing mitochondrial tagged DJ-1 protected the human neuroblastoma cell line SK-N-BE-2-C against ROS to a greater extent than wild-type DJ-1 or nucleus tagged DJ-1 [28]. In both cases, DJ-1 localized to the mitochondrial outer membrane [27,28]. Permeabilization of the mitochondrial membrane is critical for the initiation and execution of cell death signaling [28]. DJ-1 is also present in the inter membrane space and matrix of the mitochondria [30]. Nutrient starvation promotes DJ-1 mitochondrial translocation [31]. C106T, M26I, and L166P DJ-1 mutants fail to translocate to the mitochondria upon nutrient starvation [31]. These studies highlight DJ-1 mitochondrial localization following stimulation, but the precise location and role of various stimuli remain to be determined. Taken together, these observations suggest DJ-1 may serve important roles in mitochondrial function and subsequent cell survival.

In primary cortical neurons and mouse embryonic fibroblasts, DJ-1 deficiency or knockdown is associated with altered mitochondrial morphology, including mitochondrial length, fragmentation, and fusion and/or fission processes [32,33,34]. This is also observed in vivo in the striatum of DJ-1-deficient mice and mutant L166P DJ-1 cells [31,32]. Treatment of DJ-1-deficient primary cortical neurons with the ROS scavenger NAC did not affect mitochondrial length but attenuated mitochondrial fragmentation [19]. Overexpression of wild-type DJ-1 and not the C106A DJ-1 mutant rescued the aberrant mitochondrial phenotype in DJ-1-deficient neurons [32]. In another study, mitochondrial matrix-targeted DJ-1 rescued and promoted mitochondrial elongation. Loss of DJ-1 did not alter the expression of cytochrome C oxidase IV or the outer membrane protein Tom20, in primary cortical neurons [32]. DJ-1 deficiency in dopaminergic neurons did not affect the expression of mitochondrial subunits except for COX4, a subunit of complex IV [35]. Loss of DJ-1 also impaired Complex I assembly [35]. In addition to morphological/structural changes, loss of DJ-1 or mutations in DJ-1 alter mitochondrial function. DJ-1−/− mouse embryonic fibroblasts display multiple mitochondrial impairments including functional and morphological changes: (i) an increase of mitochondrial ROS formation in DJ-1−/− cells that could impair the oxidative phosphorylation as indicated by (ii) diminished pyruvate dependent respiration rates and (iii) reduced mitochondrial membrane potential. These metabolically detectable impairments are paralleled with morphological changes such as decreased mitochondrial branching [34]. Impaired cellular bioenergetics following the loss of DJ-1, as assessed by oxygen consumption rate and extracellular acidification rates, has been shown in multiple in vitro and in vivo investigations [32,34]. Mitochondrial membrane potential is also reduced in DJ-1 knockout mouse embryonic fibroblasts [34]. Analysis of transcriptome data from park7−/− zebrafish brains suggests that mitochondrial oxidative stress and iron dyshomeostasis comprise early preclinical events in PD [36].

The underlying defects in mitochondrial morphology, dynamics, and function are associated with loss of or dysfunctional DJ-1 and may determine the cellular response to stress. The role of DJ-1 in regulating mitochondrial integrity and function is two-fold: (i) oxidative stress from the loss of DJ-1 can cause mitochondrial damage leading to increased oxidative stress and upregulation of DJ-1, and (ii) mitochondrial dysfunction has been characterized as one of the most important contributors of PD [37].

## 4. Cytoprotective Activities of DJ-1

### 4.1. DJ-1 and Reactive Oxygen Species

Of the multitude of functions ascribed to DJ-1, its primary role is that of an antioxidant. Antioxidants are defined as molecules that can directly scavenge ROS, accelerate secondary ROS degrading/quenching pathways, and/or inhibit ROS generating pathways that may be related to the single function of binding to multiple mRNA transcripts [14,38,39]. DJ-1 acts as an antioxidant via all three mechanisms. The responsiveness of DJ-1 to oxidative insults was first observed with lipopolysaccharide (LPS), H_2_O_2_, or paraquat treatment. These treatments shifted the isoelectric point (pI) of DJ-1 from 6.2 to 5.8—increased oxidized (acidic) and decreased reduced (basic) DJ-1 protein expression [27,40,41,42]. Acidic isoforms were also detected in patients with idiopathic PD [43]. In various cell types (including microglia, astrocytes, neurons, airway epithelial cells), an increase in DJ-1 mRNA and protein is observed following various oxidative stimuli [24,27,41,42]. Increased oxidized DJ-1 (i.e., acidic species) was found with paraquat exposure and with increasing age in Drosophila, mouse brain, and samples of human skin [44]. Other indirect oxidative stressors, such as exposure to cigarette smoke, upregulated oxidized DJ-1 in the lung epithelial cell line Beas2b and the adeno-carcinomic human alveolar basal epithelial A549 cells [27,45,46]. In all cases, the induction of DJ-1 in response to oxidative insults served as a cellular feedback mechanism to downregulate oxidative stress. Furthermore, cells harboring mutant forms of DJ-1, including L166P, became susceptible to death in parallel with the loss of oxidized forms of DJ-1 [24]. Consistent with its antioxidant role, the genetic silencing of DJ-1 using siRNA increased ROS production and oxidative injury in a variety of cell types [27,33,47,48,49,50]. DJ-1−/− or DJ-1 knockdown mice models further showed increased ROS production in heart, brain, skeletal muscle, lung, and kidney following injury [32,51,52]. Overexpression of DJ-1 reversed oxidative stress conditions. Overexpression of wild-type DJ-1, and not the L166P or C106A mutant, protects cells against oxidative insults [7,24,27,33,53,54,55]. DJ-1 also exhibited peroxiredoxin (antioxidant)-like activities in controlling cytokine-induced peroxide levels [47]. 

The cytoprotective effects of DJ-1 against oxidative stress depend on its cysteine residues. Cysteine oxidation is an important post-translational modification that quenches free cellular ROS. Human and mouse DJ-1 have three cysteine residues at positions 46, 53, and 106, of which cys-106 is the most prominent in regulating oxidative stress [27]. Evidence supporting Cys106 as the critical residue of DJ-1 and the favored target of modification comes from studies demonstrating that this residue is intolerant to mutagenesis compared to the other cysteine [37]. The mutation of Cys106 to serine, alanine, or aspartic acid abolishes the antioxidant capacity of DJ-1 [56]. Cys106 can be oxidized either to sulfinate (-SO_2_) or to sulfonate (-SO_3_) with differing effects on protein stability and conformation [56]. Cys106-SO_2_ is more stable, whereas Cys106-SO_3_ may clash with surrounding residues and alter the DJ-1 structure. This biphasic response of Cys106 to oxidation allows DJ-1 to recognize levels of oxidative stress and respond accordingly. In human umbilical vein endothelial cells treated with hydrogen peroxide, Cys106 was completely oxidized to the sulfonate (Cys-106 SO_3_) [48]. Cys106 interacts with a neighboring protonated Glu18 residue, stabilizing the Cys106-SO_2_-(sulfinic acid) form of DJ-1. Glu18 mutations (E18N, E18D, E18Q) alter the oxidative propensity of Cys106 with enhanced susceptibility of Cys106 to oxidation in E18N and E18A DJ-1 but a reduced propensity for cysteine oxidation in E18D DJ-1 [57]. Thus, supporting the hypothesis that other non-cysteine and non-methionine residues play important contributory roles in the antioxidant capacity of DJ-1. Several studies have proposed that DJ-1 in its native (reduced) form can scavenge ROS. In response to moderative oxidative stress, the formation of DJ-1 SO_2_- (DJ-1 sulfinate) assumes a protective role in enhancing mitochondrial localization or binding and inhibiting cell death initiators. Following excessive oxidative stress conditions, the conversion of DJ-1 to DJ-1 SO_3_- (DJ-1 sulfonate) leads to aberrant DJ-1 aggregation, loss of its antioxidant, antiapoptotic, and cytoprotective functions [37,58]. The latter case appears to be reminiscent of features of PD as supported by the presence of extensively oxidized cysteine and methionine residues in post-mortem brains of sporadic PD and Alzheimer’s disease patients [59]. Furthermore, crystallography and mass spectrometry studies showed that DJ-1 sulfonate is highly unstable [19]. Mutagenesis of C106A prevented DJ-1 instability following oxidation and increasing the dose and length of oxidant exposure reduced DJ-1 [60]. Moreover, in either A549 cells treated with H_2_O_2_ or M17 neuroblastoma cells treated with paraquat, the recovery of DJ-1 from an oxidized to reduced DJ-1 form after removal of stimuli took 6–24 h; recovery was completely blocked in the presence of cycloheximide (CHX, a de novo protein synthesis inhibitor), demonstrating new protein synthesis is required for recovery [27,45].

DJ-1 is a positive regulator of Nrf2, a key transcription factor regulating a battery of cellular antioxidant genes and maintaining cellular redox status. DJ-1 stabilizes Nrf2 by impairing Keap1-mediated degradation of Nrf2 [61]. DJ-1knockdown in lung small cell carcinoma cells decreased the Nrf2 half-life and expression of the Nrf2 target genes NQO1 and GCLM [61]. Similarly, siRNA DJ-1 knockdown in mice and cell lines (Beas2b and mouse embryonic fibroblasts) conferred Nrf2 protein instability and reduced induction of Nrf2 dependent genes (NQO1, GCLM, HMOX1) [60]. Furthermore, DJ-1 knockdown in human corneal endothelial cells irradiated with UV-A decreased Nrf2, HMOX1, and NQO1 expression [62]. Moreover, loss of DJ-1 reduced Nrf2-dependent antioxidants in the lungs from chronic obstructive pulmonary disease patients [60]. DJ-1 may also negatively modulate ROS-generating molecules. DJ-1 silencing via siRNA in renal proximal tubule cells increased ROS production and NADPH oxidase 4 (NOX4) expression and activity [63]. In mice, deletion of DJ-1 in renal cells resulted in diminished Nrf2 expression and oxidative stress-dependent hypertension [20]. Experimental studies have also demonstrated that DJ-1 stimulates Nrf2 translocation to the nucleus end enhances its recruitment to the thioredoxin1 (Trx1) promoter [64]. The DJ-1, Nrf2, and STING (stimulator of interferon genes) pathways are also targeted by the anti-PD naturally occurring compound Withaferin A [65].

Overall, DJ-1 appears to mediate its antioxidant effects through both direct and indirect mechanisms. It has intrinsic activity as an atypical peroxiredoxin-like peroxidase that scavenges free reactive oxygen radicals. It also appears to positively regulate Nrf2-induced antioxidant pathways. Missense mutations in DJ-1 reduce its ability to function as an antioxidant. These findings suggest that DJ-1 acts as a sensor of cellular redox status and responds appropriately with various cytoprotective signaling pathways.

### 4.2. DJ-1 and Cell Death

DJ-1′s protective role in preventing cell death has been established; mutation or loss of DJ-1 leads to cell death. The absence of DJ-1 in baseline conditions (i.e., without any challenge) does not show any effect on cell death [66]. The most consistent findings in DJ-1’s protective role have been observed in primary cells and transformed cell lines from the brain following various ROS-inducing agents [67]. The absence of DJ-1 combined with the presence of these neurotoxins results in increased susceptibility to oxidative stress-induced apoptotic cell death; these findings have been replicated in multiple studies in dopaminergic neurons, cortical neurons, cerebellar granule neurons, neuro-2A (mouse neuroblastoma cell line), SHSY5Y (human neuroblastoma cell line), primary microglia, BV-2 (microglial cell line), and astrocytes [7,24,52,68,69,70]. In addition, treatment with direct ROS compounds like H_2_O_2_ or indirectly via ROS-inducing stressors, such as hypoxia, similarly led to enhanced cell death in multiple cell types, including MEFs, HeLa, COS-7, Beas-2b, and cardiomyocytes [51,62,71]. Various markers and assays of cell death, including MTT assay, LDH assay, TUNEL assay, Annexin V, caspase-3 cleavage, and PARP cleavage, all show greater cell death in DJ-1-deficient cells and tissues following stimulation [24,62,70,72,73]. Moreover, this hypersensitivity to oxidative stress translated to cortical and dopaminergic neuron loss and behavioral abnormalities in DJ-1-deficient mice in in vivo models of PD [52,69,70,74,75,76]. 

Overexpression or restoration of wild-type DJ-1 mediated via adenoviral vector delivery rescued cells from cell death. Overexpression of DJ-1 in mesencephalic dopaminergic neurons (MN9D) and COS-7 cells reduced Annexin V and TUNEL staining and increased cell viability by MTT compared to controls following treatment with the insecticide rotenone and the prodrug to the neurotoxin MPP+, which causes permanent symptoms of Parkinson’s disease [70,72]. DJ-1 overexpression using adenovirus in the substantia nigra of rats attenuated rotenone-induced cell death of dopaminergic neurons [35]. Moreover, this attenuation also reduced behavioral symptoms associated with PD [70,72]. DJ-1 overexpressing astrocytes protected against rotenone-induced cell death [62,77]. Overexpression of DJ-1 in alveolar type II cells treated with cigarette smoke extract attenuated apoptosis [16]. Both transient and stable transfection of wild-type DJ-1 in DJ-1−/− MEFs conferred protection following H_2_O_2_ exposure, as assessed by LDH assay [73]. Overexpression of DJ-1 upregulates many cell-protective signaling pathways, including ERK1/2; protection is eliminated when pretreated with a MEK1/2 inhibitor (U0126) [72,78]. This protective effect was further lost with overexpression of experimental mutant DJ-1 C106A and clinically relevant mutant DJ-1s, such as L166P, D149A, and M26I [7,73,78], suggesting mutations of DJ-1 may potentiate oxidative stress-induced cell death in PD patients. The role of DJ-1 in cell death in the setting of non-oxidative insults does not seem as essential; treatment of cortical neurons with the topoisomerase poison camptothecin and the protein kinase inhibitor staurosporine, for instance, did not increase susceptibility to cell death, implying its predominant role in cellular oxidant defense and/or its response to selective initiators of damage [68,70]. In addition to attenuating cell death indirectly by scavenging cellular ROS, DJ-1 also prevents cell death by directly binding to mediators of the apoptosis pathway. 

Under oxidative stress conditions, DJ-1 directly interacts with nuclear death domain-associated protein (Daxx) to prevent cytoplasmic translocation and binding to apoptosis signal-regulating kinase 1 (ASK1), delaying apoptosis [20,79]. In another study, DJ-1 directly bound to ASK1 in HEK293 cells following H_2_O_2_ treatment [73]. The C106A and the triple cysteine mutant (cysteine to alanine) DJ-1mutants failed to bind to ASK1 [73], revealing the role of Cys106 oxidation during ASK1 binding. Alternatively, the clinical mutant M26I DJ-1 bound much more readily to ASK1 but failed to repress ASK1 activity and cytoplasmic translocation of Daxx [73]. DJ-1 directly interacts with homeodomain-interacting protein kinase 1 (HIPK1); HIPK1 regulates both the Daxx/ASK1 pathway and p53 pathway [80]. DJ-1 upregulates cell survival signaling pathways, including AKT [29]. In low to moderate oxidative conditions (0–100 μM H_2_O_2_), the endoplasmic reticulum regulator SG2NA interacts with DJ-1 and AKT at the plasma membrane to confer protection; however, this protective activity failed under excessive oxidative stress (>100 μM H_2_O_2_) [29].

Under oxidative stress, DJ-1 downregulates Bax and protects against cell death by inhibiting the transcriptional activity of the tumor suppressor p53 on the Bax promoter [81,82]. Oxidized DJ-1 inhibits p53 by sequestering p53 from promoters in a DNA-binding affinity-dependent manner [83]. Another study demonstrated that DJ-1 activates p53 as a response to oxidative stress and then p53 decreases DJ-1 through a negative feedback loop [84]. Moreover, DJ-1 was reported to suppress p53 activity through binding with SIRT1 [85]. Intestinal DJ-1 expression was reduced in patients with inflammatory bowel disease and low DJ-1 expression aggravated p53-mediated intestinal epithelial cell apoptosis in mice with induced colitis [86]. In colorectal cancer, DJ-1 is found to indirectly reduce p53 expression, promoting cancer cell proliferation [87]. 

These findings suggest that DJ-1 prevents cell death via several mechanisms, including (i) elimination of ROS, (ii) preventing activation of cell death machinery, and (iii) upregulation of pro-survival signaling pathways. However, overwhelming oxidative stress impairs DJ-1 function and the ability to protect against cell death.

### 4.3. DJ-1 and Autophagy

Given its role in PD, many studies have investigated DJ-1’s autophagic role in neuron cell lines and brain tissues of animals, revealing that DJ-1 has a role in regulating autophagy. However, the response of DJ-1 to autophagy appears to be inconsistent. DJ-1 positively and negatively regulates autophagy in different cell lines and tissues. 

DJ-1 regulates autophagy through the JNK/Beclin1 signaling axis [66]. Over expression of DJ-1 represses autophagy by inhibiting the conversion of LC3-I to LC3-II as shown in A549 (adenocarcinoma human alveolar basal epithelial cell lines) and H1299 (p53 deficient cell line), HeLa cells, HEK293, and the neuroblastoma cell line SHSY5Y [66]. siRNA-mediated DJ-1 knockdown in the above cell lines increased LC3-II levels and LC3 punctate formation [66]. DJ-1 knockdown further decreased p62 and increased Beclin1 mRNA and protein levels [66]. The L166P DJ-1 mutant does not regulate autophagy [66]. The SH-SY5Y cell line is unresponsive to DJ-1 knockdown [66]. The authors concluded that the observed differential responses of DJ-1 may relate to the function of autophagy in cancers versus PD [66]. In the human osteosarcoma cell line U2OS, DJ-1 modulated the activity of the phosphatidylinositol 3-kinase-related kinase family member mTOR during normoxic and hypoxic conditions [71]. DJ-1 knockdown using siRNA reduced phosphorylation of the phosphatidylinositol 3-kinase-related kinase family member mTOR substrates p70-S6K and 4E-BP1 under both normoxia and hypoxia conditions [71]. DJ-1 deficiency decreased the phosphorylation of the serine/threonine kinase Akt an upstream positive regulator of mTOR. LC3 conversion from LC3-I to LC3-II was augmented in DJ-1-deficient cells compared with controls at normoxia; hypoxia increased LC3 conversion in control cells but not in DJ-1-deficient cells, implying a defect in autophagy following cellular stress [71]. Appropriate turnover of p62 by autophagy (referred to as autophagy flux) is required to avoid abnormal aggregate formation. High levels of p62 persisted in hypoxic DJ-1-expressing U2OS cells [71].

In another study investigating autophagy, DJ-1−/−MEFs showed lower levels of p62 and LC3-II compared to WT [32]. This reduction can be attributed to the downregulation of autophagy or enhanced autophagic degradation. Using Bafilomycin A, the authors recovered p62 and LC3-II levels in DJ-1−/− MEFs, suggesting that the loss of DJ-1 enhanced autophagy flux (i.e., overactive autophagy) [32]. Another study similarly showed decreased p62 and LC3-II in DJ-1−/−MEFs that were restored with the addition of lysosomal protease inhibitors [34]. Selective DJ-1 overexpression in the mitochondrial matrix, as opposed to the cytosol, promoted autophagy flux [31]. 

These findings suggest that localization of autophagic processes in the mitochondria may be more protective. The differences observed in the various studies in the role of DJ-1 loss in autophagy induction and blockage may be attributed to various cell types and treatments across the different studies. More investigations are needed to evaluate the role of DJ-1 in the regulation of autophagic processes.

### 4.4. DJ-1 and Inflammation

There is an emerging focus on DJ-1’s role in immunity. Some studies have established DJ-1’s role in modulating adaptive immune responses. In a rodent model of passive cutaneous anaphylaxis, DJ-1-deficient mice had altered IgE mediated allergic responses. The loss of DJ-1 increased levels of ROS, TNF, IL-4, mast cell recruitment, and degranulation of mast cells [88]. In DJ-1-deficient bone marrow-derived mast cells, the phosphorylation of the tyrosine-protein kinase Syk was suppressed but that of the linker for activation of T cells (LAT) was enhanced [88]. Furthermore, phosphorylation of PLCγ and the MAP kinases Erk1/2, JNK, and p38 were all augmented in DJ-1-deficient mast cells [88]. Stromal cell-derived factor (SDF)-1, a T cell chemokine, and its receptor CXCR4 are involved in T cell migration and proliferation. DJ-1-deficient mice had enhanced CD3+ T cell migration and CXCR4 expression with increasing concentrations of SDF-1 [89]. Treatment with a CXCR4 inhibitor normalized these differences. Furthermore, in DJ-1-deficient mice, the CD3+ T cell subsets Th1 and Th17 had increased production of IFN-γ and IL-17 [90]. CD3 and CXCR4 expression was also enhanced in the neointima formed in the ligated artery in DJ-1−/− mice [89]. Regulatory T cells (CD4+CD25+Foxp3+) are pivotal for adaptive immunity and maintenance of thymic and peripheral self-tolerance, and for protection against collateral damage induced during excessive inflammation. Regulatory T cells were more abundant in the thymus and periphery of DJ-1−/− mice [44]. DJ-1 also affects innate immunity. DJ-1−/− *Caenorhabditis elegans* develop marked p38 mitogen-activated protein kinase activation and display enhanced pattern recognition protein expression when grown on pathogenic *Pseudomonas aeruginosa* [90]. Increased respiratory burst occurs in DJ-1−/− *Litopenaeus vannamei* (white leg shrimp) following *Vibrio alginolyticus* (a marine gram-negative bacteria) challenge [91].

Several studies investigating the role of DJ-1 in the brain have revealed its role in regulating inflammation. Following LPS treatment, DJ-1-deficient astrocytes had increased TNF levels, iNOS, NO, IL-6, cyclooxygenase-2, and p38 phosphorylation [46,50]. Similarly, DJ-1’s anti-inflammatory role was demonstrated in DJ-1-deficient astrocytes and microglia that exhibited increased expression of TNF, cyclooxygenase-2, and iNOS and increased STAT1 phosphorylation following IFN-γ treatment [74]. Cortical brain slices from DJ-1−/− mice following IFN-γ treatment showed enhanced IL-6 and TNF levels and STAT1 phosphorylation [74]. In another study, LPS administration in the substantia-nigra of DJ-1−/− mice increased ICAM-1, IFN-γ, IL-1β, IL-16, IL-17, and I-TAC(CXCL11) levels [69]. Additionally, DJ-1 knockdown promoted early phosphorylation of IKK and IkBα resulting in increased p65 nuclear translocation under both basal and LPS-treated conditions [69]. The high NF-κB promoter activity in DJ-1 knockdown cells further demonstrates the negative regulatory role of DJ-1 in neuroinflammation [69]. 

**Figure 2:**
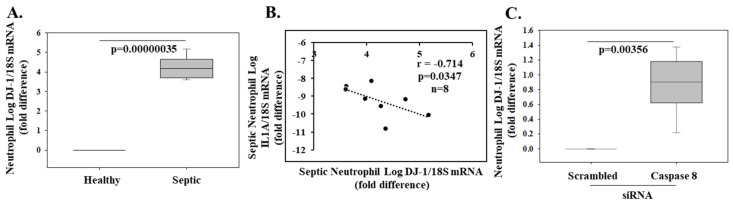
Polymorphonuclear cell DJ-1 expression is increased in septic patients and inversely correlates with the inflammatory cytokine ILA. Neutrophil RNA was isolated from healthy controls (*n* = 3) and septic patients (*n* = 8) using TRIzol reagent and reverse transcribed into cDNA. DJ-1, IL1A, and 18S (housekeeping) gene expression was determined by real-time quantitative (RT-PCR). The Human Ethics Review Committee of St. Michael’s Hospital reviewed and approved the ‘inflammation in trauma and sepsis’ (INSIST) study protocol for collecting blood from healthy volunteers and septic patients. Written informed consent was obtained from patients or a surrogate decision-maker. DJ-1/18S and 1L1A/18S mRNA expression in septic neutrophils was calculated as a log-fold change over healthy controls and presented as box plots showing the median and interquartile range (**A**). Negative correlation between pro-inflammatory IL1A and DJ-1 (**B**). Human septic neutrophils were transfected with a scrambled (control) or a caspase 8 (apical caspase in the extrinsic cell death pathway) siRNA for 24 h (**C**).

DJ-1-deficient mice exhibited greater susceptibility to LPS-induced acute lung injury as demonstrated by increased cellular infiltration, augmented levels of pulmonary cytokines, enhanced ROS levels and oxidized by-products, increased pulmonary edema, and cell death. In a two-hit model of LPS and mechanical ventilation, DJ-1-deficient mice displayed enhanced susceptibility to inflammation and lung injury. Collectively, these results identify DJ-1 as a negative regulator of ROS and inflammation, and suggest its expression protects from sterile lung injury driven by high oxidative stress [92]. In direct contrast to the above-mentioned studies, DJ-1−/−mice were protected against cecal ligation and puncture (CLP)-induced polymicrobial sepsis. DJ-1 deficiency enhanced pro-inflammatory cytokine production and bacterial clearance in different organs and blood. The absence of DJ-1 was further associated with increased phagocytosis and bacterial killing via higher NADPH oxidase 2 activity [93]. The conflicting data can be explained by the divergent role of DJ-1 in bacterial clearance versus inflammation. In a model without live bacteria, i.e., endotoxin only, the overzealous pro-inflammatory response has no benefit and only leads to collateral tissue damage. This adds to the litany of previous evidence of pro-inflammatory cytokine knockout mice showing variable outcomes depending on the experimental sepsis model utilized. These findings further add to our understanding of distinct host outcomes to infectious versus sterile inflammation insults. To complement our review, we conducted qPCR studies in primary human neutrophils from patients with sepsis [94] as described in the Supplementary Materials and Methods online. Our data demonstrated higher DJ-1 mRNA in 8 septic patients (2 male and 6 female; mean age 69.3 ± 11.0 years; multiorgan dysfunction score 7.3 ± 2.7; WBC 24.3 ± 13.6) when normalized to neutrophils from healthy controls (*p* = 0.00000035) (Figure 2A). There was a negative correlation between DJ-1 and pro-inflammatory IL-1A gene expression (r = −0.714; *p* = 0.0347; *n* = 8) (Figure 2B). Moreover, in septic neutrophils, siRNA silencing of caspase 8, a member of the extrinsic cell death pathway, further increased DJ-1 mRNA (Figure 2C).

Together these findings suggest that DJ-1’s role in regulating the inflammatory response is complex and requires further investigation. 

## 5. Cardioprotective Function

Considering DJ-1’s antioxidant properties and the role of oxidative stress in heart disease, a limited number of studies were conducted to investigate a possible association. 

Acute preconditioning is an endogenous mechanism through which brief, reversible ischemic episodes reduce myocyte necrosis after prolonged coronary occlusion. Chronic preconditioning persists for up to 4 days and reduces infarct size while protecting the heart from ischemia-induced myocardial stunning. Myocardial postconditioning is a protective mechanism engaged via intermittent ischemia at the time of reperfusion, occurring through the activation of reperfusion injury kinase pathways that limit the opening of the mitochondrial permeability transition pore [95].

DJ-1 overexpression in HL-1 cardiac cells reduced cell death from simulated ischemia-reperfusion injury and delayed mitochondrial permeability transition pore opening, a critical mediator of cell death. Exposure of H9c2 cells derived from embryonic rat heart to hypoxic preconditioning and 24 h later to hypoxia/reoxygenation upregulated DJ-1, reduced ROS production and increased cell viability whereas the beneficial effects of preconditioning were largely diminished in cells transfected with siRNA against DJ-1. These results implicate that DJ-1 is involved in the delayed cardio-protection of ischemic preconditioning [96]. The same group of researchers demonstrated that DJ-1 promotes delayed cardio-protection after hypoxic preconditioning through the activation of Nrf-2 and upregulation of antioxidant enzymes [97]. Although DJ-1 levels were reduced in H9c2 cells subjected to ischemia-reperfusion, transfection of pIRES2-DJ-1 and subsequent overexpression of DJ-1 attenuated ROS production and cell apoptosis. The proposed mechanism was a DJ-1-mediated reduction of pro-apoptotic PTEN and promotion of the PI3K/Akt signaling pathway [98]. Similarly, DJ-1 expression was upregulated in rat myocardium after ischemic preconditioning in vivo while delayed cardio-protection of preconditioning was attenuated in DJ-1 knockdown rats [99]. Interestingly, hearts from DJ-1 wild type and knock-out mice exhibited no differences in left ventricular dimensions and systolic function under baseline conditions [100]. DJ-1 also attenuates myocardial ischemia-reperfusion injury via regulation of mitochondrial fission [44].

**Figure 3:**
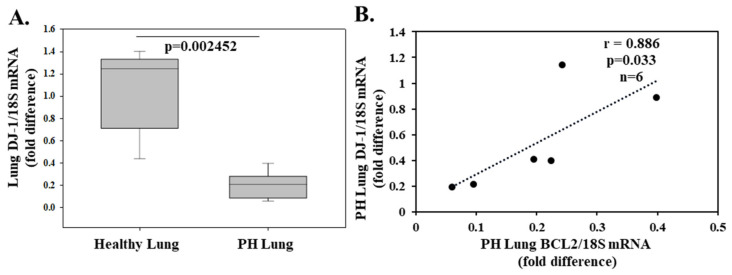
Lung DJ-1 expression is decreased in patients with pulmonary hypertension (PH). In a prospective study, 12 patients of whom 6 (4 male and 2 female; mean ± SEM age 59.8 ± 4.4 years) were cancer patients negative for lung malignancy with a pulmonary artery systolic pressure <25 mmHg and 6 (4 male and 2 female; mean age 64.5 ± 3.1 years) with thromboembolic pulmonary hypertension negative for malignancy with a mean ± SEM pulmonary artery systolic pressure of 70 ± 4 mmHg. The lung biopsies were taken from the left lower lobe by video-assisted thoracic surgery. The pulmonary artery systolic pressure was assessed using echocardiography measuring maximal tricuspid regurgitation velocity and applying the modified Bernoulli equation to convert this value into pressure. The study was approved by the ethics committee of the University of Athens General Hospital Attikon, and informed consent was obtained from all patients. DJ-1, BCL2, and 18S (housekeeping) gene expression in the lung was determined by real-time quantitative (RT-PCR). DJ-1/18S mRNA in PH was calculated as a fold change over healthy and presented as box plots showing the median and interquartile range. p value shown (**A**). Positive correlation between anti-apoptotic BCL2 and DJ-1 in PH patients (**B**).

Hypertension affects more than a billion people worldwide, and its pathophysiology comprises a variety of neurohormonal, vascular, renal, and genetic mechanisms. Evidence from experimental and clinical studies shows an association between hypertension and oxidative stress implicating a causal relationship [101]. The renal dopamine 2 receptor (D2R) controls the pathogenesis of hypertension and, in fact, mice lacking the D2R develop ROS-dependent hypertension. Cuevas et al. [66] reported that kidney DJ-1 expression is regulated by D2R and in part mediates its antioxidant effects. Silencing of renal DJ-1 expression resulted in ROS production and increased systolic blood pressure [67]. In another study, DJ-1 deletion increased norepinephrine-induced vasoconstriction and reduced acetylcholine-induced relaxation in endothelium-intact mice aortic rings. Moreover, endothelial cells from DJ-1−/− mice demonstrated higher levels of H_2_O_2_ and decreased NO production [102]. The same group reported neointimal plaque formation after carotid artery ligation was greater in DJ-1−/− mice, implicating that DJ-1 contributes to vascular smooth muscle cell growth [102]. In a rat model of hypoxia-induced pulmonary arterial hypertension, DJ-1 was decreased in rat lung. Similarly, decreased DJ-1 was detected in hypoxia-induced pulmonary artery smooth muscle cells. Furthermore, knockout of DJ-1 aggravated hypoxic pulmonary arterial hypertension in rats [103]. To complement the experimental results, we conducted qPCR studies in lung biopsies from cancer patients (negative for malignancy) and from patients with thromboembolic pulmonary hypertension (negative for malignancy) as described in the Supplementary Materials and Methods online. We show that lung DJ-1 expression is decreased in 6 patients (4 male and 2 female; mean age 64.5 ± 3.1 years) with thromboembolic pulmonary hypertension with a mean ± SEM pulmonary artery systolic pressure of 70 ± 4 mmHg compared to 6 cancer patients (4 male and 2 female; mean ± SEM age 59.8 ± 4.4 years) with a pulmonary artery systolic pressure <25 mmHg (Figure 3A). Furthermore, highlighting the anti-apoptotic effect of DJ-1, we observed a positive correlation between DJ-1 and anti-apoptotic BCL2 in lung biopsies from pulmonary hypertensive patients (r = 0.886, *p* = 0.033, *n* = 6; Figure 3B) thereby, making DJ-1 an attractive therapeutic target in the treatment of pulmonary hypertension. 

Some studies investigated DJ-1’s role in metabolic syndrome. After a lifestyle intervention comprising diet and exercise instructions in Japanese women, a decrease in blood pressure, blood glucose levels, and body mass index was observed compared to controls and was associated with a concomitant increase in DJ-1 levels following intervention [104]. Moreover, DJ-1 was found to positively regulate LDL receptor expression in mice, implicating a role for DJ-1 in cholesterol metabolism. LDL levels were increased in DJ-1 knockout male mice compared to controls, but not in female mice [104].

**Figure 4:**
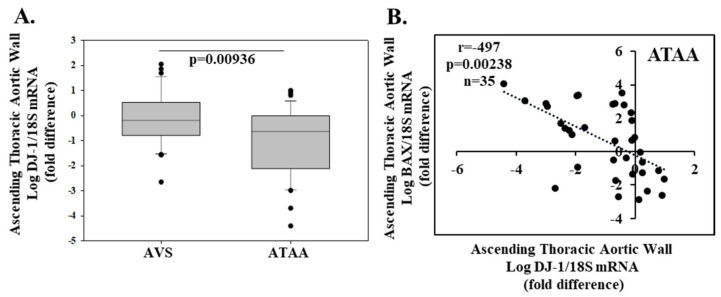
Aortic wall DJ-1 expression is decreased in patients with ascending thoracic aneurism. In a prospective study, 66 consecutive patients of whom 31 (18 male and 13 female; mean age 64.6 ± 11.5 years) with normal ascending thoracic aortic diameter underwent atrial valve replacement for severe aortic valve stenosis (AVS), and 35 patients (28 male and 7 female; mean age 65 ± 10.2 years) underwent replacement of an ascending thoracic aortic aneurysm (ATAA). The study was approved by the ethics committee of the University of Athens General Hospital Attikon, and informed consent was obtained from all patients. Biopsy-sized pieces of the aortic wall were collected from the greater curvature of the distal aortic root neighboring the anterolateral portion of the sino-tubular junction (ATAA). DJ-1, BAX, and 18S (housekeeping) gene expression was determined by real-time quantitative (RT-PCR). DJ-1/18S mRNA in ATAA was calculated as fold change over AVS and presented as box plots showing the median and interquartile range. p value shown (**A**). Negative correlation between pro-apoptotic BAX and DJ-1 in ATAA patients (**B**).

There are no studies investigating the role of DJ-1 in ascending thoracic aortic aneurysm (ATAA). In novel data generated for this review using a patient population recently described [105] and methodology available online, we show that thoracic aortic wall DJ-1 expression is decreased in 35 patients (28 male and 7 female; mean ± SEM age 65 ± 10.2 years) undergoing replacement for an ATAA compared to thoracic aortic DJ-1 expression in 31 (18 male and 13 female; mean age 64.6 ± 11.5 years) patients with normal ascending thoracic aortic diameter undergoing aortic valve replacement for severe aortic valve stenosis (AVS) (Figure 4A). In keeping with the ani-apoptotic effect of DJ-1, we observed a negative correlation between DJ-1 and the pro-apoptotic marker BAX in the ascending thoracic aorta of ATAA patients (r = −0.497, *p* = 0.00238, *n* = 35; Figure 4B). These observations suggest the possibility that the level of DJ-1 may become a novel anti-apoptotic marker in ATAA patients.

Heart failure is a complex syndrome defined as the inability of the heart to pump blood effectively for oxygen demands or only being able to do so at the expense of increased filling pressures. The onset may be abrupt (such as after myocardial infarction) or progressive. Irrespective of this, various systems contribute to the pathophysiology of heart failure, including a sympathetic nervous system, the renin-angiotensin system, neurohormonal alterations of renal function, and altered cardiac myocyte biology, a process known as LV remodeling [95]. The involvement of oxidative stress in the development and progression of heart failure has been recognized [95]. Both experimental evidence and data from heart failure patients report increased ROS production and diminished activity of the major antioxidant systems compared to the controls, leading to oxidative stress. Although pre-clinical trials with anti-oxidative stress treatments in animals were promising, clinical trials failed to show significant benefit [59]. Yet therapies that increase endogenous antioxidant capacity in heart failure continue to be proposed as future targets [86]. Apart from its role in redox homeostasis, DJ-1 regulates apoptosis and autophagy. DJ-1expression declined in the process of pressure overload cardiac hypertrophy in parallel with impaired autophagy. DJ-1 knockout mice exhibited a more hypertrophied phenotype than wild-type, indicating DJ-1 is responsible for the repression of cardiac hypertrophy [106]. DJ-1 knockout mice subjected to pressure overload via transaortic banding exhibited enhanced pathologic cardiac hypertrophy, increased interstitial fibrosis, and reduced fractional shortening compared to controls. No differences in cardiac phenotype were observed between groups under normal conditions. The authors also measured DJ-1 levels from left ventricular samples of end-stage heart failure patients and healthy controls and showed DJ-1 was reduced in human patients with end-stage heart failure [51]. DJ-1 knockout mice subjected to ischemia-reperfusion exhibited enhanced left ventricular dilatation, myocyte hypertrophy, and fibrosis and reduced systolic function compared to control. Overexpression of DJ-1 attenuated the adverse effects of ischemia reperfusion [51]. The authors concluded that DJ-1 protects against ischemia-reperfusion-induced heart failure partly by modulating glycative stress [107]. In the same study, samples from end-stage heart failure patients exhibited lower DJ-1 levels and increased AGEs and RAGEs compared to healthy controls [107]. Similarly, DJ-1 levels increased post-coronary artery bypass grafting (CABG) in non-diabetic patients compared to pre-CABG levels while the AGEs/sRAGEs ratio was decreased post-CABG [108], both suggesting that DJ-1 has a role in the post-CABG improvement of cardiac biology and implying the involvement of DJ-1 in the regulation of glycative stress. These observations are in line with previous evidence that suggested a deglycase activity of DJ-1. Experimental evidence has shown that DJ-1 exhibits protein deglycase activity, repairing methylglyoxal- and glyoxal-glycated cysteine, arginine, and lysine residues [109]. DJ-1 also repairs nucleotides in a similar manner, before their irreversible conversion into AGEs [15]. Moreover, DJ-1 overexpression diminished the methylglyoxal-induced glycation and aggregation of the brain protein a-synuclein [110], which plays a crucial role in the pathophysiology of PD.

**Figure 5:**
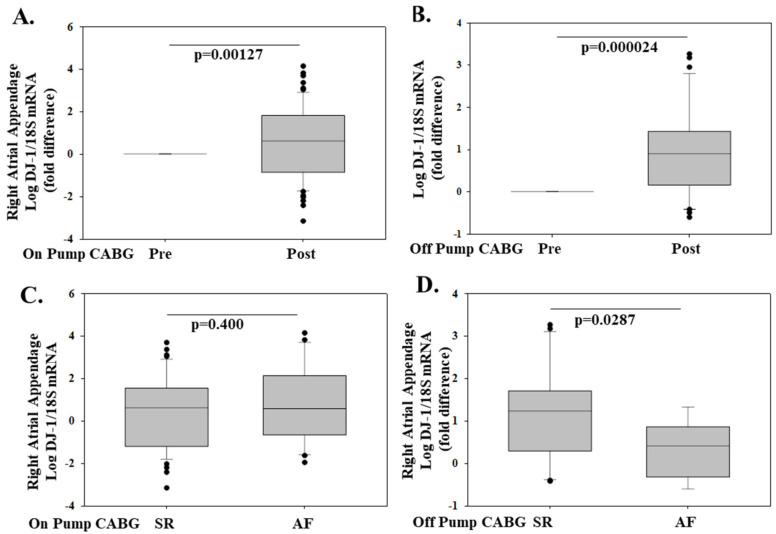
Right atrial appendage (RAA) DJ-1 mRNA is increased post-on and -off CABG specifically in off-pump patients that remain in sinus rhythm (SR). This prospective study examined 67 patients (44 males and 23 females, mean age 64.1 ± 1.13 years) that underwent elective on-pump coronary artery bypass grafting (CABG) and 31 patients (28 male and 3 females, mean age 64.1 ± 1.53 years) that underwent elective off-pump surgery. Inclusion criteria were as follows: (1) preoperative SR; (2) elective CABG. Exclusion criteria were as follows: (1) history of AF/flutter; (2) history of prior cardiac surgery; and any antiarrhythmic medication pre or peri-operatively. Post-operative atrial fibrillation (POAF) was based on the documentation of AF episodes (>30 s) by continuous ECG monitoring up to 7 days after surgery. The study was approved by the ethics committee of the University of Athens General Hospital Attikon, and informed consent was obtained from all patients. RAA was obtained from the same location pre and post CABG. DJ-1 and 18S (housekeeping) gene expression was determined by real-time quantitative (RT-PCR). RAA DJ-1/18S mRNA is represented as a fold change versus pre-CABG (**A**,**B**) and post-CABG patients that remained in SR or developed atrial fibrillation AF (**C**,**D**) are presented as box plots showing the median and interquartile range. *p* values are shown.

Using a patient population recently described [111], and methodology available is the online, we show that right atrial appendage (RAA) DJ-1 mRNA is increased post-CABG compared to pre-CABG levels in 67 patients (44 males and 23 females, mean age 64.1 ± 1.13 years) that underwent elective on-pump (CABG) and in 31 patients (28 male and 3 females, mean age 64.1 ± 1.53 years) that underwent elective off-pump surgery in the (Figure 5A,B). In on-pump patients, post-CABG RAA DJ-1 expression was similar in patients that remained in sinus rhythm (SR) compared to patients that developed post-operative atrial fibrillation (POAF) (Figure 5C). Interestingly, in off-pump patients, post-CABG RAA DJ-1 was lower in patients that developed POAF compared to patients that remained in SR (Figure 5D). Preoperative DJ-1 might be a biomarker that could offer superior risk stratification of patients undergoing major elective CABG surgery either on-pump or off-pump with respect to POAF development and is worthy of further investigation as a potential therapeutic target. 

## 6. Concluding Remarks and Future Implications

**Figure 6:**
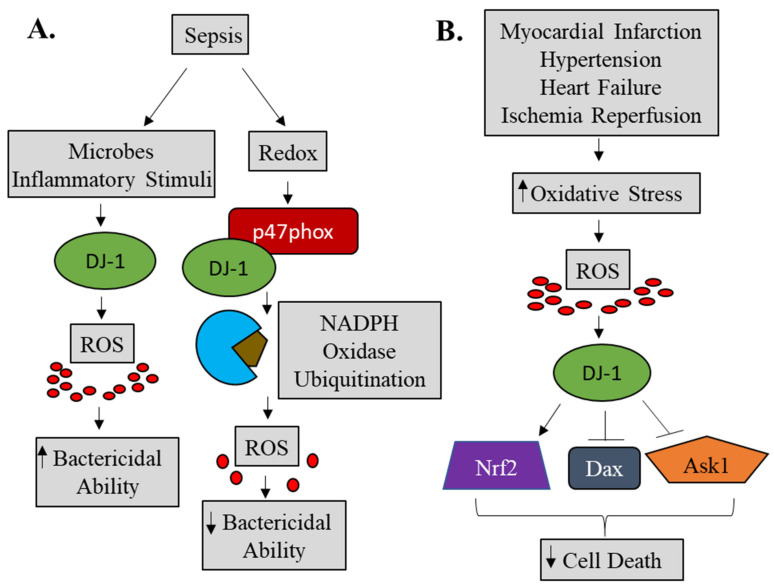
Schematic of DJ-1’s potential role in sepsis and cardiovascular disease. Our data are summarized in this schematic diagram. In innate immune cells, following an inflammatory or bacterial stimulus, DJ-1 expression is increased. Available DJ-1 can regulate bactericidal activity by directly scavenging superoxide ions and reducing ROS. Alternatively, in the redox state, DJ-1 may bind to p47phox and disrupt NADPH oxidase complex assembly, or ubiquitinate NADPH oxidase, subsequently leading to decreased ROS production (**A**). In response to an increase in oxidative stress, DJ-1 can exert antioxidant function by upregulating Nrf2 activation and downregulating pro-apoptotic Dax and Ask1 (**B**). Nrf2, NF-E2 related factor-2; ASK1, apoptosis signal-regulating kinase 1; Daxx, death-associated protein 6.

DJ-1, a protein originally described as a novel oncogene and afterward associated with early-onset Parkinson’s disease, plays a much more complicated role than originally recognized. Our novel and published data [92,93] concerning DJ-1 and sepsis suggests that DJ-1 exerts protective functions in the active state of macrophages through ROS production to facilitate the killing of bacteria whereas, in the redox state, DJ-1 can reduce ROS through binding to p47^phox^, thereby, disrupting downstream signaling pathways (Figure 6A). Taken together, the functional role of DJ-1 in sepsis remains complex, and further studies are warranted if DJ-1 is to become a potential therapeutic target. 

Although the role of oxidative stress is well established in cardiovascular diseases such as ischemia and heart failure and implicated in others (atrial fibrillation, hypertension, aortic aneurysm), the functional role for DJ-1 remains less clear. The available experimental evidence suggests a possible cardio-protective function of DJ-1 that may involve upregulating antioxidant gene expression, through the transcription factor Nrf2, and downregulating apoptotic signaling pathways (Figure 6B). Indeed, our novel data suggests a potential anti-apoptotic role of DJ-1 in ATAA, PH, and CABG patients. 

Two human studies have already associated low levels of DJ-1 with heart failure, and we now report an increase of DJ-1 in post-CABG ischemic heart disease compared to pre-CABG. Although most clinical studies using antioxidant therapies have failed to demonstrate a significant benefit, DJ-1 not only has a pivotal role in redox homeostasis but exhibits substantial beneficial functions that could enhance its favorable effect on cardiac function. However, there is a series of questions that need to be answered. First, the correlation of DJ-1 levels in peripheral blood and cardiac tissue needs to be established. Further studies should confirm the beneficial effects of DJ-1 in vivo, and then interventions that could increase DJ-1 levels should be evaluated. Taking into consideration its multifunctional properties, we hypothesize that DJ-1 is a promising target for intervention against cardiovascular disease, and, therefore, further research is warranted in this direction. 

## Data Availability

Data presented in manuscript is original data. Additional data available upon request. All figures used in the manuscript are original and there is no copyright issue.

## Supplementary Materials

Materials and Methods are available online.

## Abbreviations

A	alanine
AGEs	advanced glycation end products
ASK1	apoptosis signal-regulating kinase 1
ATAA	ascending thoracic aortic aneurysm
AVS	aortic valve stenosis
A549	adenocarcinomic human alveolar basal epithelial cell line 549
α	alpha
β	beta
Bax	Bcl2-associated X protein
Bcl2	B-cell lymphoma 2
Beas2b	bronchial epithelium cell line
BV-2	microglial cell line
C	cysteine
CABG	coronary artery bypass grafting
CD	cluster of differentiation
CHX	cycloheximide
CLP	cecal ligation and puncture
COS-7	CV-1 in origin with SV40 genes cell line
COX4	cytochrome oxidase subunit 4
C-terminal	carboxy-terminal
CXCR4	CXC chemokine receptor 4
Cys	cysteine
D	aspartic acid
Daxx	death domain-associated protein
D2R	dopamine 2 receptor
DJ-1−/−	DJ-1 knockout
E	glutamic acid
ERK	extracellular signal-regulated kinase
Foxp3	forkhead box P3
GCLM	glutamate-cysteine ligase modifier subunit
Glu	glutamic acid
HEK293	human embryonic kidney cell line 293
HeLA	Henrietta Lacks (cancer cell line)
HIPK1	homeodomain-interacting protein kinase 1
HMOX1	heme oxygenase 1
H1299	p53 deficient cell line
H_2_O_2_	hydrogen peroxide
HL-1	cardiac cell line
H9c2	rat ventricular cell line
I	isoleucine
ICAM-1	intercellular adhesion molecule 1
IFNγ	interferon gamma
IgE	immunoglobulin E
IKK	inhibitor of nuclear factor kappa B kinase
IkBα	nuclear factor of kappa light polypeptide gene enhancer in B-cells inhibitor,
IL-1A	interleukin 1 alpha
IL-1β	interleukin 1 beta
IL-4	interleukin 4
IL-6	interleukin 6
IL-16	interleukin 16
IL-17	interleukin 17
iNOS	inducible nitric oxide synthase
INSIST	inflammation in trauma and sepsis
IRES	internal ribosomal entry site
I-TAC	interferon-inducible T-cell alpha chemoattractant
JNK	Janus kinase
K	lysine
Keap-1	kelch-like ECH-associated protein 1
kDa	kilodalton
L	leucine
LAT	linker for activation of T cells
LC3	microtubule-associated protein light chain 3
LDH	lactate dehydrogenase
LDL	Low-density lipoprotein
LPS	lipopolysaccaride
LV	left ventricle
M	methionine
MAP	mitogen-activated protein
MEF	mouse embryonic fibroblasts
mmHg	millimeter of mercury
MN9D	mesencephalic dopaminergic neurons
MPP+	1-methyl-4-phenylpyridinium
mTOR	mammalian target of rapamycin
MTT	3-(4,5-dimethylthiazol-2-yl)-2,5-diphenyltetrazolium bromide
mRNA	messenger RNA
M17	neuroblastoma cells
N	asparagine
NAC	N-acetyl cysteine
NADPH	nicotinamide adenine dinucleotide phosphate
Neuro-2A	mouse neuroblastoma cell line
NF-κB	nuclear factor kappa-light-chain-enhancer of activated B cells
NO	nitric oxide
NQO1	NAD(P)H quinone oxidoreductase 1
Nrf2	NF-E2 related factor-2
NOX	NADPH oxidase
NOX4	NADPH oxidase 4
P	proline
PARK7	Parkinson’s disease-associated gene 7
PARP	poly (ADP-ribose) polymerase
PD	Parkinson’s disease
PH	pulmonary hypertension
pI	isoelectric point
PI3K	phosphoinositide 3 kinase
PLCγ	phospholipase C gamma
POAF	post-operative atrial fibrillation
PTEN	phosphatase and tensin homolog
Q	glutamine
qPCR	quantitative polymerase chain reaction
RAA	right atrial appendage
RAGE	receptor for advanced glycation endproducts
ROS	reactive oxygen species
SDF-1	stromal cell derived factor
SEM	standard error of the mean
SG2NA	S/G2 nuclear autoantigen
SHSY5Y	human neuroblastoma cell line
siRNA	small interfering ribonucleic acid
SIRT1	sirtuin 1
SK-N-BE-2C	human neuroblastoma cell line
SR	sinus rhythm
STAT1	signal transducer and activator of transcription 1
Syk	spleen tyrosine kinase
SO_2_	sulfinate
SO_3_	sulfonate
T	threonine
TNF	tumor necrosis factor
Tom20	translocase of outer mitochondrial membrane 20
Trx1	thioredoxin 1
Th1	T helper 1
Th17	T helper 17
μM	micromolar
U2O2	human bone osteosarcoma epithelial cell line
WBC	white blood cell
WT	wild type
