# Supplementary material for: Cytoprotective Mechanisms of DJ-1: Implications in Cardiac Pathophysiology
Source: Molecules. 2021 Jun 22;26(13):3795. doi: 10.3390/molecules26133795 (PMC8270312; doi:10.3390/molecules26133795)
Supplement: Supplementary file 1 [file molecules-26-03795-s001.zip › molecules-1239111-supplementary-corected.pdf]

molecules

MDPI

Supplementary Material

Cytoprotective Mechanisms of DJ-1: Implications in Cardiac Pathophysiology

James N. Tsoporis [1,*,†], Ioannis-Alexandros Drosatos [2,†], Sahil Gupta [1,3,†], Hajera Amatullah [4], Shehla Izhar [1], Claudia C. dos Santos [1,3], Vasileos Salpeas [2], Angelos G. Rigopoulos [2], Ioannis K. Toumpoulis [2], Andreas S. Triantafyllis [2], Eleftharios Sakadakis [2], Nikolaos Kavantzas [5], John C. Marshall [1,3], Ioannis K. Rizos [2,†], and Thomas G. Parker [1,†]

[1] Keenan Research Centre for Biomedical Science, Li Ka Shing Knowledge Institute, St. Michael's Hospital, University of Toronto, Ontario, M5B 1W8, Canada; Sahil.Gupta@unityhealth.to (S.G.); shehla.izhar@unityhealth.to (S.I.); claudia.dossantos@unityhealth.to (C.C.d.S.); john.marshall@unityhealth.to (J.C.M.); thomas.parker@unityhealth.to (T.G.P.)
[2] 2nd Academic Department of Cardiology, Attikon University Hospital, University of Athens Medical School, Athens, 164 62, Greece; alexdrosatos@hotmail.com (I.-A.D.); vsalpeas@med.uoa.gr (V.S.); angelos.rigopoulos@gmail.com (A.G.R.); toumpoul@otenet.gr (I.K.T.); andreas.triantafyllis@gmail.com (A.S.T.); elsakadakis@yahoo.gr (E.S.); ioannis.c.rizos@otenet.gr (I.K.R.)
[3] Institute of Medical Science, University of Toronto, Ontario, M5G 1V7, Canada
[4] Gastrointestinal Unit and Center for the Study of Inflammatory Bowel Disease, Massachusetts General Hospital and Harvard Medical School, Boston, MA, 02210, USA; hajera.amatullah@gmail.com
[5] 1st Department of Pathology, School of Medicine, National and Kapodistrian, University of Athens, Athens, 115 27, Greece; nkavantz@med.uoa.gr
* Correspondence: jimtsoporis@sympatico.ca.
† These authors contributed equally to this work.

Citation: Tsoporis, J.N.; Drosatos, I-A.; Gupta, S.; Amatullah, H.; Izhar, S.; Santos, C.D.; Salpeas, V.; Rigopoulos, A.G.; Toumpoulis, I.K.; Triantafyllis, A.S.; et al. Cytoprotective mechanisms of DJ-1: Implications in cardiac pathophysiology. Molecules 2021, 26, 3795. https://doi.org/10.3390/molecules26133795

Academic Editors: Diego Muñoz-Torrero; Simona Rapposeli; Michael Gütschow; Maria João Matos; Maria Emília de Sousa and Luciano Saso

Received: 12 May 2021
Accepted: 11 June 2021
Published: 22 June 2021

Publisher's Note: MDPI stays neutral with regard to jurisdictional claims in published maps and institutional affiliations.

Copyright: © 2021 by the authors. Submitted for possible open access publication under the terms and conditions of the Creative Commons Attribution (CC BY) license (http://creativecommons.org/licenses/by/4.0/).

Supplemental Methodology

Clinical data

1. Septic Patients

The Human Ethics Review Committee of St. Michael's Hospital reviewed and approved the 'inflammation in trauma and sepsis' (INSIST) study protocol (research ethic board protocol # 12/216) for collecting blood from healthy volunteers and septic patients. Written informed consent was obtained from patients or a surrogate decision-maker. A sub-population of septic patients from the INSIST study (2 male and 6 female; mean age 69.3 ± 11.0 years; multiorgan dysfunction score 7.3 ± 2.7; WBC 24.3 ± 13.6) was compared with 6 healthy volunteers (2 male and 6 female; mean age 65.4 ± 8.5 years). Peripheral blood neutrophils were isolated from blood as described below.

2. Pulmonary Hypertension Patients

Archival blocks of formalin-fixed, paraffin-embedded heart tissue from 6 decreased patients (4 male and 2 female; mean age 64.5 ± 3.1 years) with thromboembolic pulmonary hypertension with a mean ± SEM pulmonary artery systolic pressure of 70 ± 4 mmHg, were compared to 6 cancer patients (4 male and 2 female; mean ± SEM age 59.8 ± 4.4 years) negative for lung malignancy with a pulmonary artery systolic pressure <25 mmHg were obtained through a collaboration (research ethic board protocol # 10/2021) with 1st Department of Pathology, School of Medicine, National and Kapodistrian, University of Athens, Greece.

3. Ascending Thoracic Aortic Aneurysm Patients

This prospective study examined 66 consecutive patients of whom 31 (18 male and 13 female; mean age 64.6 ± 11.5 years) underwent AVR for severe AVS, who had a normal

ascending thoracic aortic diameter (all patients with AVS had tricuspid aortic valves) and 35 patients (28 male and 7 female; mean age 65 ± 10.2 years) who underwent replacement of an ATAA [2]. The study was approved by the ethics committee and informed consent was obtained from all patients. Before the initiation of any study procedures, written informed consent was obtained from each study participant. The ethics committee of our institution (National and Kapostrian University of Athens Medical School, Attikon University Hospital, Athens, Greece) approved the study protocol (# 210/21). Biopsy-sized pieces of the aortic wall were collected from the greater curvature of the distal aortic root in proximity to the anterolateral portion of the sino-tubular junction (ATAA), frozen in liquid nitrogen and stored at −80 °C until analysis.

4. On- and Off-Pump CABG patients

This prospective study examined 67 patients (44 males and 23 females, mean age 64.1 ± 1.3 years) [3] that underwent on-pump CABG and in 31 patients (28 male and 3 females, mean age 64.1 ± 1.53 years) that underwent elective off-pump surgery. Inclusion criteria were as follows: (1) preoperative SR; (2) elective CABG. Exclusion criteria were as follows: (1) history of AF/flutter; (2) history of prior cardiac surgery; and any antiarrhythmic medication pre or peri-operatively except for β-blocking agents. Post-operative AF was based on documentation of AF episodes (>30 s) by continuous ECG monitoring up to 7 days after surgery. The study was approved (protocol # 450) by the ethics committee (National and Kapostrian University of Athens Medical School, Attikon University Hospital, Athens, Greece), and informed consent was obtained from all patients. Tissue samples of right atrial appendage (RAA) were obtained from the same location pre- and post-CABG frozen in liquid nitrogen and stored at −80°C until analysis.

Neutrophil Purification, Isolation, and Transfection

Peripheral blood neutrophils were isolated by density gradient centrifugation using Ficoll-Hypaque (GE Healthcare, Little Chalfont, UK) as previously described [1]. The neutrophils were suspended (1 × 10^6 cells/mL) in 10% autologous serum in DMEM and 1% (*v*/*v*) penicillin-streptomycin (Invitrogen, Burlingto2n, ON, Canada) in polypropylene tubes, and incubated at 37 °C in a 5% carbon dioxide atmosphere. Control neutrophils were incubated with LPS (1 µg/mL), from *Escherichia coli* O111:B4 (Sigma–Aldrich, Mississauga, ON, Canada). Septic neutrophils were transfected with siRNA directed at either caspase-8 (ThermoFisher, Mississauga, ON, Canada) or scramble control (Santa Cruz Biotechnology, Santa Cruz, CA) for 24 h using the transfection reagent LipofectamineRNAiMAX according to the instructions provided by the manufacturer (ThermoFisher).

Real-Time RTPCR

RNA was harvested from neutrophils, aorta, RAA, and paraffin-embedded heart tissue sections using the RNeasy Plus mini kit (Qiagen) according to the company's protocol. Real-time quantitative RT-PCR was performed according to the instructions of the manufacturer (Qiagen, Alameda, CA). In brief, a 25 µL reaction volume containing 12.5 µL of RT^2 SYBR Green qPCR Master Mix, 10.5 µL of H_20, 1 µl of gene-specific 10 µM PCR primer pair stock of gene of interest (human primers for 18S, DJ-1, IL1A, Bax, and Bcl2–proprietary sequences) (Qiagen, Alameda, CA) and 1 µL of RT^2 First-Strand cDNA (template) underwent a two-step cycling program, 40 cycles, 10 min at 95 °C, 15 s at 95 °C, and 1 min at 60 °C. In separate experiments, the threshold cycle (C_t) value for the housekeeping gene 18S and for the gene of interest in each sample was determined. For each sample, the difference between the C_t values (ΔCt) for each gene of interest and 18S was calculated. For each pair-wise set of samples to be compared, the differences in ΔCt values ($\Delta\Delta C_t$) for the genes of interest between the two samples were calculated. Due to the guaranteed consistently high levels of amplification efficiency across the RT-2 qPCR Primer Assays, the fold-change in gene expression is equal to $2^{(-\Delta\Delta Ct)}$. For each gene of interest in the

experimental group, the (log_2) mean fold-change is shown relative to the gene of interest in the control group.

Statistics

Depending on the class of analysed data and possible direction of causality, distributions were compared with a 2-sample t-test, Mann–Whitney Rank Sum test, or analysis of variance followed by the Student–Newman–Keuls test. Data are presented as (log_2) mean value ± standard error of the mean (SEM) for normally distributed continuous data or median (interquartile range–BOX plot) for non-normally distributed data. Spearman's coefficients of correlation were calculated between the variables. Probability values of $p <$ 0.05 were accepted as statistically significant.

References

1. Gupta, S; Lee, C.M.; Wang, J.F.; Parodo, J.; Jia, S.H.; Hu, J.; Marshall, J.C. Heat-shock protein-90 prolongs septic neutrophil survival by protecting c-Src kinase and caspase-8 from proteasomal degradation. *J. Leukoc Biol.* 2018;*103*:933–944.
2. Rizos, I,K.; Tsoporis, J.N.; Toumpoulis, I.K.; Salpeas, V.; Izhar, S.; Rigopoulos, A.G.; Sakadakis, E.A.; Parker. T.G. Antiapoptotic Effect of beta1 Blockers in Ascending Thoracic Aortic Smooth Muscle Cells: The Role of HSP70 Expression. *J. Cardiovasc Pharmacol*. 2018;*72*:86–96.
3. Tsoporis, J.N.; Fazio, A.; Rizos, I.K.; Izhar, S.; Proteau, G.;, Salpeas, V.; Rigopoulos, A.; Sakadakis, E.; Toumpoulis, I.K.; Parker, T.G. Increased right atrial appendage apoptosis is associated with differential regulation of candidate MicroRNAs 1 and 133A in patients who developed atrial fibrillation after cardiac surgery. *J. Mol. Cell Cardiol.* 2018;*121*:25-32.
